# The Impact of Obesity on Perioperative Outcomes for Children Undergoing Appendectomy for Acute Appendicitis: A Systematic Review

**DOI:** 10.3390/jcm12144811

**Published:** 2023-07-21

**Authors:** Nikolaos Zavras, Natalia Vaou, Sofia Zouganeli, Arezina Kasti, Papakonstantinou Dimitrios, George Vaos

**Affiliations:** 1 Department of Pediatric Surgery, Medical School, National and Kapodistrian University of Athens, Attikon University General Hospital, 12462 Athens, Greece; gvaos@med.uoa.gr; 2 Department of Nutrition and Dietetics, Attikon University General Hospital, 12462 Athens, Greece; szoug@yahoo.co.uk (S.Z.); kastiare@yahoo.gr (A.K.); 3 Third Department of Surgery, Attikon University General Hospital, 12462 Athens, Greece; dimpapa7@hotmail.com

**Keywords:** children, obesity, acute appendicitis, appendectomy, perioperative outcomes, postoperative complications

## Abstract

Today, the prevalence of obesity in the pediatric population has increased dramatically. Acute appendicitis (AA) is the most common surgical condition among pediatric patients. We aimed to investigate the impact of obesity on postoperative outcomes in terms of operative time (OT), length of stay (LOS), surgical site infection (SSI), overall complications, adverse events, and mortality in children undergoing appendectomy for acute appendicitis. An extensive search of the literature in PubMed and Google Scholar was conducted to evaluate the outcomes of normal weight (NW), overweight (OW), and obese (OB) children who underwent appendectomy. Although no statistically significant differences were noted in perioperative outcomes and overall postoperative complications between OW/OB and NW children in the majority of the included studies, prolonged OT and LOS and SSI were found in some studies. Moreover, no differences in terms of readmissions and ED visits were recorded. We conclude that the impact of obesity on postoperative outcomes for children undergoing appendectomy for AA is unclear, and, therefore, no safe conclusions can be drawn with the currently available data. Due to the lack of high-quality studies, further research is required to optimize the surgical approach and prevent unwarranted complications.

## 1. Introduction

Over the last three decades, a dramatic increase in obesity among children and adolescents in the United States and developed countries has resulted in a major worldwide public health problem [1]. According to existing evidence on obesity, 80% of adolescents aged 10–14 years, 50% of children aged 6–9 years, and 25% of children under the age of 5 have an increased possibility of remaining obese as adults [2]. Similarly, the prevalence of overweight (OW) and obese (OB) pediatric surgical patients has increased [3].

Pediatric obesity is a multifactorial entity carrying complications and health risks that involve a diversity of affected organ systems, such as the cardiovascular system (hypertension, left ventricular hypertrophy, and atherosclerosis), pulmonary system (obstructive sleep apnea and asthma), gastrointestinal system (gastroesophageal reflux and non-alcoholic fatty liver disease), skeletal system (slipped capital-femoral epiphysis and tibia vera), and metabolic system (insulin resistance, diabetes type 2, and dyslipidemia) [4]. In addition, childhood obesity and obesity-related diseases not only impact immediate health outcomes but also carry psychological side effects and social and economic consequences and negatively impact long-term morbidity and mortality [5,6,7,8].

Perioperative studies in adults and children have shown that obese patients are prone to anesthetic complications due to a difficult airway, impeded mask ventilation and direct laryngoscopy, protracted stay in the post-anesthesia care unit, increased antiemetic use, and more frequent unexpected overnight hospitalizations and hospital readmissions [9,10,11]. In addition, childhood obesity may mislead the diagnosis of common pediatric disorders such as acute appendicitis (AA), thereby increasing cases of unnecessary appendectomies, or may even worsen postoperative outcomes in children undergoing adenotonsillectomy [12,13,14]. The aim of this systematic review is to investigate the impact of obesity on perioperative outcomes for children undergoing appendectomy for AA. To the best of our knowledge, this is the first systematic review to investigate the impact of obesity on perioperative outcomes in children with AA.

## 2. Materials and Methods

### 2.1. Study Protocol

This systematic review is reported in accordance with the Preferred Reporting Items for Systematic Reviews and Meta-analysis (PRISMA) [15] and was registered in the international prospective register of systematic reviews (ID: CRD42023423574).

### 2.2. Inclusion and Exclusion Criteria

Selection criteria included original clinical studies of prospective or retrospective design, i.e., randomized and non-randomized trials and prospective observational and retrospective studies with primary data on postoperative complications, readmissions, and mortality for AA in OW/OB children compared to their normal weight counterparts.

We excluded studies pertaining to bariatric surgery and studies that did not have clinical information on the type of treatment, perioperative outcomes, and postoperative complications. Non-clinical articles such as conference abstracts, editorials, case reports, any reviews, and unavailable full-text articles and articles not written in the English language were also excluded.

### 2.3. Search Strategy

We searched three major databases (PubMed, Science Direct, and Scopus) for English-language full-text articles published from 1 January 2007 to 1 May 2023. In addition, Google Scholar was selected for the gray literature search. Two authors (NZ and NV) performed all searches and stored them in EndNote, X6 For Windows. Our search was supplemented by harvesting references from the bibliography of included studies to ensure a thorough literature review.

Searches were conducted using the keywords “children”, “adolescents”, “pediatric surgery operations”, “perioperative outcomes”, “postoperative complications”, “hospital readmissions”, “reoperations”, and “emergency department visits”. Specifically, we used the following search terms and medical subject headings: (“obesity” OR “children” OR “adolescents”) AND (“acute appendicitis” OR “perioperative outcomes” OR “postoperative complications” OR “postoperative adverse events”). Duplicate titles were removed by EndNote^TM^.

### 2.4. Study Screening

Two authors (NZ and NV) independently screened the titles and abstracts of the article list generated by the search algorithm. In cases of disagreement, a solution by common consensus was attempted. A senior author (GV) verified the accuracy of the collection process and resolved any remaining disagreements. All identified full-text publications were read in full by two authors (NV and NZ). After the removal of duplicated articles and articles that fell within the exclusion criteria, all remaining articles were reviewed by three independent authors (NZ, NV, and GV), and any disputes were also resolved by consensus.

### 2.5. Data Items

Data extraction included publication details, study design, and patient characteristics. The collected data were classified into three categories: (1) preoperative data: demographic and anthropometric data, underlying disease, and surgery settings; (2) operative data: type of surgery (open or laparoscopic techniques), conversion to open surgery, and operative time (OT); and (3) postoperative data: length of stay (LOS), surgical site infection (SSI), overall complications, readmissions, reoperations, or emergency department (ED) visits within 30 days of discharge, and mortality. Primary outcomes of interest were OT, LOS, SSI (as defined by each study), and overall postoperative complications. Secondary outcomes included readmissions, reoperations, or emergency department visits within 30 days of discharge and in-hospital mortality. Outcomes were recorded at any point in time, i.e., both in-hospital and post-discharge if available. All data were extracted and entered into standardized excel spreadsheets (Microsoft, Redmond, WA, USA) for further data tabulation.

### 2.6. Definitions

Expert committee recommendations/(Centers for Disease Control and Prevention (CDC) classification): According to these recommendations, the body mass index (BMI) in children and adolescents between 2 and 18 years of age is defined as follows: (1) underweight (UW): a BMI < the 5th percentile for age; (2) normal weight (NW): a BMI between the 5th and 84th percentile; (3) overweight (OW): a BMI between the 85th and 94th percentile; and (4) obese (OB): a BMI ≥ the 95th percentile for age [16].

WHO classification: the definition of overweight and obesity in childhood is based on pooled international data for body mass index and linked to the widely used adult obesity cut-off point of 30 kg/m^2^ [17].

Any postoperative complications within 30 days of the initial procedure were recorded. Complications were stratified as medical and surgical. Medical complications involved postoperative pneumonia, urinary tract infection, renal impairment or acute renal failure, deep vein thrombosis, and unplanned intubation or requirement of mechanical ventilation. Surgical complications included any surgical site infection (SSI) and wound class as defined according to the USA CDC criteria [18]. Specifically, surgical complications were categorized according to the Clavien–Dindo classification. Clavien 0: no complications. Clavien I: any deviation from the normal postoperative course without the need for pharmacological or other intervention treatment. Clavien II: any complication necessitating drugs other than such allowed for grade I complications. Clavien III: complications that call for surgical, endoscopic, or radiological interventions. Clavien IV: life-threatening complications that require intermediate or intensive care management. Clavien V: death of the patient. Clavien I–II are grouped as minor complications. Clavien III–V are grouped as major complications [19].

## 3. Results

### 3.1. Study Selection

**Figure 1:**
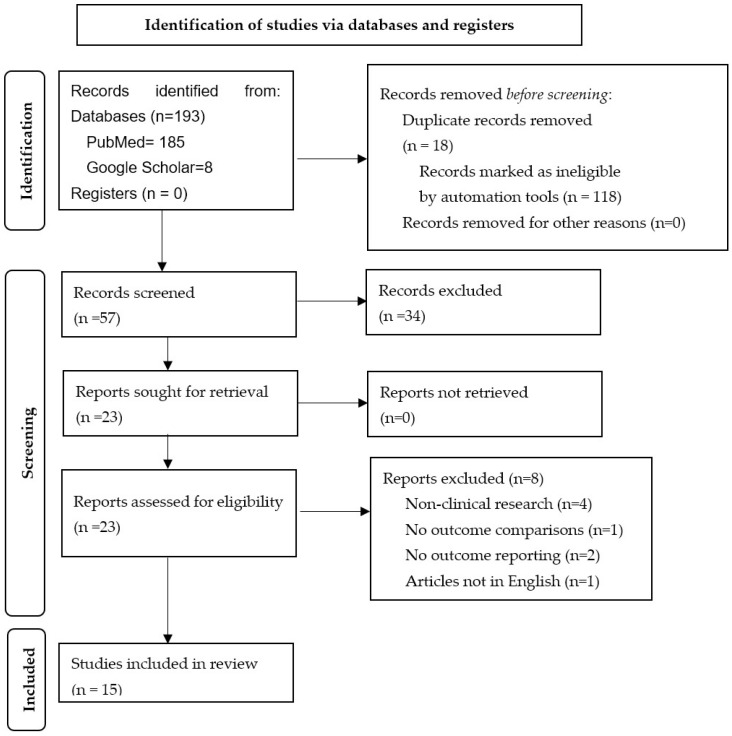
PRISMA flow-chart describing study screening and inclusion.

The initial database search yielded 145 articles. Duplicate records (n = 15) were removed, and records were organized according to titles, abstracts, and full reports (n = 107). The main exclusion reasons upon full-text screening were non-clinical research (n = 4), no outcome comparisons (n = 2), and no outcome reporting (n = 2). The final synthesis included 15 full-text articles that met the predetermined inclusion and exclusion criteria. The PRISMA flow-chart details the study inclusion (Figure 1).

### 3.2. Studies’ Characteristics and Patient Demographics

Basic publication details and patient demographics are presented in Table 1. Included studies [20,21,22,23,24,25,26,27,28,29,30,31,32,33,34] were published between 2007 and 2023. The smallest study included 94 patients, and the largest included 9606 patients. Study designs consisted of nine retrospective studies [20,21,24,27,28,29,30,31,33], four prospective studies [23,25,26,32], and one meta-analysis [22]. The majority (n = 12) of the studies were derived from a single-center institution, [20,21,22,23,24,25,26,28,29,31,32], while 2 studies included patients from a national database [27,30]. Of the 14 studies, 9 were conducted in the USA [22,24,25,26,27,29,30,31,33], 2 in Canada [20,23], and 1 each in Austria [21], the Netherlands [29], and Spain [32].

### 3.3. Perioperative Outcomes and Postoperative Complications

Surgical perioperative outcomes including the type of surgery, conversions to open surgery, OT, LOS, SSI, and postoperative complications with main results are shown in Table 2. A total of 16.140 children underwent appendectomy, either for uncomplicated (UA) or complicated (CA) appendicitis. From the existing data, it came up that the sum of overweight and obese children was 5209 (male/female ratio: 1:07), representing 32.3% of the total patients. BMI classification was carried out based on the CDC classification in 10 studies [21,22,23,24,25,26,27,28,29,30], while the rest followed the WHO classification [32] or their institutions [20,23,33]. A total of 14.822 children underwent urgent/emergency appendectomy, while the remaining 1318 were in an elective setting.

#### 3.3.1. Type of Surgery and Conversions

In eight (53.3%) studies [21,22,25,26,27,29,31,34], the conventional laparoscopy (CL) or the single-port (SP) laparoscopic technique was performed. Four studies [20,28,30,32] reported either open or laparoscopic approaches, while in three studies [23,24,32], the surgical approach was not mentioned. Based on available data, conversions, either from CL to the open method [27] or SP to CL [26,31], were reported only in these three studies.

#### 3.3.2. Operative Time

Statistically longer OT between NW versus OW/OB patients was recorded in five (33.3%) studies [20,22,27,30,32] and in a cohort study [25] including a sample of patients on whom the SP technique was exclusively performed. Moreover, Papillon et al. [34] noticed that although obesity has no impact on OT (*p* = 0.463), intraoperative time was longer in non-white-race patients (*p* < 0.001) and older children (*p* = 0.012). However, the authors did not find a possible explanation.

#### 3.3.3. Length of Stay

LOS was found to be statistically prolonged in obese patients in four (26.6%) studies [20,22,28,30]. It is notable to say that Timmerman et al. [28] observed higher LOS when comparing NW versus UW patients (*p* = 0.001). The authors speculated reasons for the higher incidence of postoperative complication rates observed in UW patients. In addition, Papillion et al. [34] reported significantly longer LOS in younger patients (*p* = 0.019), regardless of their BMI.

#### 3.3.4. Surgical Site Infection

SSI in terms of intra-abdominal abscess, wound disruption, wound infection, and dehiscence was statistically higher in OW/OB patients versus NW in 3 (20%) out of 15 studies (*p* = 0.01, *p* = 0.03, and *p* < 0.001, respectively) [22,30,32]. Notably, Witt et al. [30] observed that SSI was most common in children with chronic diseases such as esophagitis, gastritis, and asthma or congenital malformations.

#### 3.3.5. Overall Complications Rates

The overall complication rates were statistically significant only in two (13.3%) studies [28,30]. Remarkably, the overall complication rates in the study of Timmerman et al. [28] involved UW patients when compared to NW (*p* = 0.041).

#### 3.3.6. Readmissions and Emergency Department Visits within 30 Days

In 10 studies [20,21,22,24,25,28,31,32,33,34], no information was reported regarding readmissions and emergency department visits within 30 days. However, reports from five [23,26,27,29,30] studies did not show statistically significant differences regarding adverse events within 30 days postoperatively between NW and OW/OB children.

#### 3.3.7. In-Hospital Mortality

Mortality was reported in one study, with only one death [30] and no statistically significant differences among NW/OW/OB/MOB (NW: 0.02%, OW: 0.00%, OB: 0.00%, and MOB: 0.00%; *p* = 1.0).

## 4. Discussion

### 4.1. Main Messages

The impact of obesity on postoperative outcomes for children undergoing appendectomy for AA is unclear. Our findings demonstrate that an increased BMI affects OT in 33.3% of the studies, LOS in 26.6%, SSI in 20%, and overall complication rates in 13.3%. From available data [23,26,27,29,30,34] on adverse events occurring within 30 days postoperatively, there were no statistically significant differences between NW and OW/OB patients. Moreover, the results of this pooled analysis highlight the potential benefits of laparoscopic appendectomy in obese children, as in the majority of the cases (60%), a laparoscopic appendectomy, either CL or SP, was performed exclusively.

### 4.2. Primary Outcomes

OT is determined by patient-related factors, surgical and anesthetic team factors, and, most importantly, the type of surgery. Obesity constitutes a unique challenge to the surgeon in gaining access to the abdominal cavity since the abdominal wall is thicker, thus making the process of organizing the operative field a more laborious process [35]. For instance, Kutasy et al. [36] investigated the impact of CL appendectomy on OT in non-obese and very obese children. Unsurprisingly, they observed that obesity was associated with prolonged OT (45.8 versus 51.1 min, *p* < 0.005). These results are in line with the results of Garey et al. [22] and Witt et al. [30] who compared OT in NW and obese children and found that CL appendectomy is associated with statistically significantly lower OT in NW patients (*p* = 0.003 and *p* < 0.001, respectively). In contrast, Michailidou et al. [27] found longer OT in obese children only in uncomplicated AA cases (*p* = 0.004). Moreover, the introduction of the SP technique as an alternative technique to CL [21,25,31] did not demonstrate improvements in the outcomes of obese children in terms of OT. Possible reasons could be the limited degree of manipulation of the ileo-cecal region, specifically if the appendix is located behind the cecum, and the limited degree of manipulation of the working instruments through a single port [37]. Regarding laparoscopic appendectomy versus open surgery, Kutasy et al. [38], when comparing CL versus the open technique in very obese children, found significantly lower OT in the laparoscopic group (46.8 min versus 59.87 min, respectively, *p* < 0.05). Studies in obese adults who underwent laparoscopic appendectomy have shown that laparoscopy enables better visualization of the surgical field, leading to the assumption that laparoscopic appendectomy is superior to open surgery regarding OT [39]. However, the results from adult AA literature are conflicting. For instance, Enochsson et al. [40] and Hussein et al. [41] reported longer OT in the laparoscopically treated cases of AA versus the open counterparts, while three studies [39,42,43] including a meta-analysis [42] exhibited significantly shorter OT in the laparoscopic treatment groups of AA in adult obese patients. These discrepancies may be due to pathophysiological changes such as cardiac and pulmonary impairments, which are observed in obese adults or may reflect a variable experience operating on obese patients [44].

A few studies in the adult AA literature have investigated the impact of BMI on LOS in patients who underwent appendectomy [33,45]. Lorio et al. [33] reported no statistically significant differences regarding LOS in a cohort sample of 118 (obese *n* = 45, non-obese *n* = 73) patients (79.6 ± 65.5 h vs. 101.6 ± 123.0 h, respectively; *p* = 0.21). Recently, Benk et al. [45] evaluated the risks of prospective operation in non-obese and OW/OB patients by using the American College of Surgeons National Surgical Quality Improvement Program surgical risk calculator (ACS-NSQIP SRC). They found no statistically significant differences regarding LOS between NW, OW, and OB patients (18.0 ± 1.39, 1.84 ± 1.68, and 2.37 ± 2.14, *p* = 0.088, respectively). Furthermore, the results of several adult studies comparing open versus laparoscopic appendectomy in obese patients are conflicting. For example, Towfigh et al. [46] reported equivalent postoperative LOS after laparoscopic appendectomy when compared to open appendectomy (1 vs. 2 days, respectively, *p* = 0.235). In addition, Clarke et al. [47], in a prospective, randomized double-blind study, found comparable outcomes regarding LOS in obese patients who underwent either laparoscopic or open surgery (mean LOS in days: obese = 4, non-obese = 2, *p* = 0.140). However, contradictory results have been previously published in several studies [39,41,48,49]. Regarding the pediatric population, the present study indicated that only four of the eligible studies [20,22,28,30] displayed statistically significant findings (*p* = 0.048, *p* < 0.001, *p* < 0.001, and *p* = 0.002, respectively).

SSI is the most common complication affecting all kinds of surgeries and leading to prolonged hospital stays, increased medical expenses, and, finally, a negative impact on patients’ outcomes [50]. In the case of appendectomies, despite innovations with the advent of laparoscopic surgery, it still remains a procedure associated with a high risk for SSI [51]. Obesity is one of the most studied risk factors for SSI in the adult patient population [52,53]. In the pediatric population, the exact incidence of SSI after surgery in obese children remains uncertain despite the increasing prevalence of pediatric obesity. It is important to note that Blackwood et al. [54] found an elevated BMI to be a significant risk factor for SSI, with general surgery procedures being associated with the highest complication rates. Furthermore, Stey et al. [55] found that obese children ≥ the 95th weight percentile were at 1.35-fold increased odds of significant SSI. In the present study, only three (20%) studies [22,30,32] observed statistically significant SSI differences in terms of abscess formation [22], superficial wound infection [30], and wound dehiscence [32] between obese and non-obese children. This relatively low percentage could reflect the benefits of the laparoscopic approach, as most appendectomies of this review were performed laparoscopically.

### 4.3. Secondary Outcomes

Angeramo et al. [56] examined the risk factors for readmissions in obese patients undergoing appendectomy, either open or laparoscopically, and found that patient age over 50 years and/or localized peritonitis confer a higher risk of readmission and, therefore, warrant closer observation. Conversely, Bailey et al. [57], in a meta-analysis of patients undergoing appendectomy, reported that obesity is not a risk factor for readmission at 30 days after appendectomy. In this study, available data from adverse events were derived from five studies [23,26,27,29,30,34] and did not show any statistically significant differences between NW and OW/OB patients. However, the lack of cross-sectional data and the oftentimes short follow-up intervals reported in studies may distort the actual readmission rates in the general pediatric population [48]; thus, no concrete conclusions can be drawn based on existing data.

Our results showed that mortality among obese children undergoing surgery is a very infrequent event. Instead, death typically depends on the severity of existing comorbidities.

### 4.4. Emerging Challenges

Worthy of note are three important issues that emerged from the results of this study. The first relates to the definition of BMI in children and adolescents. Our results showed that there was no standard BMI definition; institutional [20,23] or national [32] approaches were applied. Such discrepancies highlight the need for a universally accepted classification of BMI in children and adolescents. The second issue concerns UW patients. The results of this study are conflicting, as Timermann et al. [28] reported that UW patients face prolongations of the LOS and increased risks of postoperative complications similar to those seen in OW/OB children, findings that are also reproduced in adult studies [58]. The third issue relates to minimally invasive surgery (MIS). Currently, MIS is applicable in up to 60% of abdominal and thoracic operations and has become an essential part of pediatric urology [59]. Data from both obese children and adults corroborated that MIS and robot-assisted laparoscopy surgery are technically feasible and safe in regard to postoperative complications, conversion rates, and LOS in OB patients [60,61]. The results of those studies are in line with the observations presented herein, suggesting that MIS in OW/OB pediatric surgical patients is safe and yields enhanced postoperative outcomes, thus making it the preferred approach for obese children.

## 5. Study Limitations

As in any systematic review, there is a possibility of missing additional views and perspectives from relevant publications, either because they were not found in our data-based search or because they were not available in the English language. Most of the publications that underwent full review were retrospective and could potentially have omitted relevant information. The majority of studies evaluating the postoperative complications of pediatric surgical procedures in OB children were of limited quality and originated from a single institution and, therefore, might not represent an accurate cross-sectional estimate. In addition, there was pronounced heterogeneity in both design and outcome reporting within the existing literature as different surgical procedures were taken into account, and valuable information such as ASA classification, comorbidities, overall complication rates, readmissions, and total hospitalization stay was often not reported. The 30-day follow-up may not suffice to gain useful information regarding the postoperative course of the patients, and long-term follow-up is warranted. Moreover, a mixed group of studies including elective, urgent, and emergent operations and a relatively small sample size of various studies were included in this systematic review. Therefore, the generalization of the results could lead to incorrect conclusions and indistinct differences.

## 6. Conclusions

The impact of obesity on postoperative outcomes for children undergoing appendectomy for AA is unclear, and, therefore, no safe conclusions can be drawn with the currently available data. Although no statistically significant differences were noted in perioperative outcomes and overall postoperative complications between OW/OB and NW children in the majority of the included studies, prolonged OT, LOS, and SSI were observed in some studies. Moreover, no differences in terms of readmission rates and ED visits were recorded. The introduction of MIS in daily practice may help overcome the technical challenges and prevent significant postoperative complications. Due to the lack of high-quality studies, further research is mandated to optimize the surgical approach and minimize postoperative complications. Although the quality of studies and the lack of standardization in reporting the outcomes can improve the surgical approach, most of the time, the choice between the classic and laparoscopic approaches depends on various factors, including the patient’s condition, the surgeon’s expertise, and available resources. A standardized set of core outcomes is needed in all acute appendicitis patients. The adoption of standardized reporting for acute appendicitis would yield beneficial effects by enabling the identification of additional risk factors for adverse outcomes beyond obesity.

## Figures and Tables

**Table 1:** Publication details and patient demographics.

Authors/Year	Country	Type of Study	Number of Patients	M/F	Age (Years or Months)	BMI Classification	Underlying Disease	Surgery Settings
Davies et al., 2007 [20]	Canada	Retrospective	273	184/98	Non-OB: 123 (mo) MOB: 122 (mo) VOB: 120 (mo)	Non-OB: 226 MOB: 25 VOB: 31 Institutional classification	Non-OB UA/CA: 164/55 MOB UA/CA: 24/0 VOB UA/CA: 24/6	Emergent: 273
Petnehasy et al., 2010 [21]	Austria	Retrospective	94	29/65	NW 12.6 (yrs) OB 12.4 yrs	NW: 65 OB: 29 CDC classification	UA/CA: 92/2	Emergent: 94
Garey et al., 2011 [22]	USA	Meta-analysis	220	130/90	Non-OB: 9.0 ± 4.4 (yrs)	Non-OB: 183 OB: 37	CA	Emergent: 220
					OB: 11.2 ± 3.7 (yrs) *p* = 0.04	CDC classification		
Sulowski et al., 2011 [23]	Canada	Prospective randomized study	263	139/124	NW: 9.9 ± 3.4 (yrs) OB: 10.5 ± 3.3 (yrs)	NW: 262 OB: 76 Institutional classification	AA	Emergent: 263
Blanco et al., 2012 [24]	USA	Retrospective	319	176/143	Non-obese 9.5 ± 3.5 (yrs) OB 8.6 ± 3.9 (yrs) *p* = 0.3	Non-OB: 257 OB: 62 CDC classification	UA Non-OB/OB: 179/34	Emergent: 319
							CA Non-OB/OB: 78/28	
Knott et al., 2012 [25]	USA	Prospective randomized study	360	192/168	NW: 11.0 ± 3.5 (yrs) OW: 10.8 ± 3.9 (yrs) OB: 12.1 ± 2.9 (yrs) *p* = 0.20	NW: 135 OW: 26 OB: 19 CDC classification	UA	Emergent: 360
Yannam et al., 2013 [26]	USA	Prospective non-randomized	500	315/185	Non-OB: 10.8 ± 4.0 (yrs) OB: 11.2 ± 3.3(yrs)	Non-OB: 395 OB: 105 CDC classification	UA Non-OB/OB: 273/80	Emergent: 411 Elective: 89
							CA Non-OB/OB: 53/5	
							IA Non-OB/OB: 69/20	
Michailidou et al., 2015 [27]	USA	Retrospective	2812	1689/1123	Non-OB: 11.3 ± 3.7 (yrs) OB: 11.0 ± 3.5 (yrs)	Non-OB: 2189 OB: 623 CDC classification	UA Non-OB/OB: 1678/454 CA Non-OB/OB: 511/169	Emergent 2812
Timmerman et al., 2016 [28]	The Netherlands	Retrospective	457	211/246	UW: 11 ± 4.07 (yrs) NW: 13.2 ± 3.51 (yrs) OW: 13 ± 3.77 (yrs) OB: 12.4 ± 3.61 (yrs)	UW: 36 NW: 346 OW: 59 OB: 16 CDC classification	AA Negative: UW: 10/36, NW: 61/346, OW: 6/59, OB: 4/16 UW vs. NW, *p* = 0.008	Urgent: 457
Litz et al., 2016 [29]	USA	Retrospective	413	N/A	NW: 11.58 ± 3.75 (yrs) OW:11.87± 3.23 (yrs) OB: 10.83± 3.53 (yrs) *p*: 0.197	NW: 274 OW: 66 OB: 73 CDC classification	UA NW: 220, OW: 54, OΒ: 62	Emergent: 413
							CA NW: 23, OW: 10, OΒ: 9	
Mohan et al., 2016 [30]	USA	Retrospective	217	123/94 *p*: 0.25	Mean age (yrs): NW: 12 OW: 13 OB:11	UW/NW: 168 OW: 30 OB: 19 CDC classification	UA: 217	Emergent: 217
Witt et al., 2016 [31]	USA	Retrospective	9606	5696/3910	Mean age (yrs): 11.07 ± 3.94	NW: 5839 OW: 1727 OB: 1572 Morbid-OB: 518 CDC classification	Appendicitis: 9606	Elective: 1193 Emergent: 5848 Urgent: 2565
Delgado-Miguel et al., 2020 [32]	Spain	Prospective non-randomized	403	249/154 *p*: 0.329	NW: 10.1 ± 3.2 (yrs) OW/OB: 10.1 ± 3.2 (yrs) *p*: 0.945	NW 306 OW/OB: 97 WHO classification	UA: 253 CA: 150	Emergent: 403
Lorio et al., 2021 [33]	USA	Retrospective	38	23/15	Mean age (yrs): Non-OB: 9 OB: 11	Non-OB/OB:29/9 Institutional classification	UA: 28 CA: 10	Emergent: 38
Papillon et al., 2023 [34]	USA	Retrospective	451	278/173	Median: 11 (yrs) (range: 9–14)	CDC classification	UA: 326 CA: 108 NA: 17	Emergent: 451

AA: Acute Appendicitis; CA, Complicated Appendicitis; CDC, Centers for Diseases Control and Prevention; IA, Interval Appendectomy; MOB, Moderately Obese; NA, Normal Appendix; NI, No Information; Non-OB, Non-obese; NW, Normal Weight; OW, Overweight; UA, Uncomplicated Appendicitis; UW, Underweight; VOB, Very Obese.

**Table 2:** Surgical perioperative outcomes and postoperative complications.

Authors	Type of Surgery	Conversions	OT (Minutes)	LOS	SSI	Overall Complications	Adverse Events within 30 Days	Mortality	Main Results
Davies et al. [20]	CL: Non-OB: 49 MOB: 5 VOB: 6	NI	Non-OB: 55 MOB: 55 VOB: 63.5 Non-OB vs. VOB (overall) *p* = 0.028	Long LOS (%) Non-OB: 23 MOB: 8.3 VOB 40 NW vs. VOB: *p*: 0.048	NW/MOB/VOB *p* = 0.07	NI	NI	NI	MOB/VOB children were associated with longer OT and LOS More common SSI in VO children
	Open: Non-OB: 170 MOB: 19 VOB: 24								
Petnehasy et al. [21]	SP	No conversion	NW: 52 (19–90) OB: 49 (27–85)	NW: 6.6 (3–14) OB: 6.6 (4–11)	NW: 1 (1%) OB: 1 (3%) *p* = NI	NW: 2 (3%) OB: 1 (3%) *p* = NSS	NI	NI	No differences regarding OT, LOS, and postoperative complications between NW and OB
Garey et al. [22]	CL	NI	Non-OB: 43.6 ± 20.5 OB: 55.2 ± 26.8 *p* = 0.003	Non-OB/OB *p* < 0.001	Abscess Non-OB/OB *p*: 0.01	NI	NI	NI	OB patients had higher rates of CA and worse outcomes
Sulowski et al. [23]	NI	NI	NI	NW (h): 23 (7–53) OB (h): 20.5 (7–54) 95% CI: 12.2 to 12	NI	NI	No statistical differences regarding ED visits between groups	NI	No major differences in outcomes between NW and OB with AA
Blanco et al. [24]	NI	NI	NI	4.14 ± 2.4 (d) vs. 4.3 ± 3.1 (d) *p*: 0.7	Non-OB/OB *p* = 0.8	NI	NI	NI	OB children had higher incidence of CA. No differences in LOS and postoperative infections
Knott et al. [25]	SP: 18 pts CL: 180 pts	NI	A. SP: NW: 34.0 ± 13.6 OB: 45.4 ± 20.1 *p*: 0.002 B. CL: NW/OW/OB *p*: 0.93	A. SP (h): NW/OW/O *p*: 0.03 B. CL: NW/OW/ OB *p*: 0.72	A. SP: NW/OW/O *p*: 0.08 B. CL NW/OW/OB *p*: 1.0	NI	NI	NI	SP appendectomy for OB children needs more OT, longer LOS, and more analgesics. No impact of CL on OB children
Yannam et al. [26]	SIPES	Conversion to CL: Non-OB: 4.1% OB: 1.9%	Non-OB: 38.9 ± 16.7 (min) OB: 40.7 ± 14.9 (min) *p* = 0.32	Non-OB: 2.3 ± 3.0 (d) OB: 2.3 ± 4.1 (d) *p* = 0.98	Wound infection Non-OB: 3.3% OB: 4.8 *p* = 0.55 Abscess Non-OB: 4.3% OB: 3.9% *p* = 0.77	Non-OB: 11.1% OB: 13.3% *p* = NI	Non-OB: 3.5% OB: 4.7% *p* = 0.56	NI	SIPES appendectomy is safe for OB children with non-increased risks for complications
Michailidou et al. [27]	CL	One conversion	UA Non-OB: 41.2 ± 23.6 OB: 44.7 ± 201 *p* = 0.004 CA Non-OB: 57.1 ± 64.8 ± 31.1 *p* = 0.016	UA Non-OB: 1.5 OB: 1.8 ± 4.3 *p* = 0.123 CA Non-OB: 5.4 ± 4 OB: 6.0 ± 4.5 *p* = 0.127	Non-OB: 23.4% Ob: 27.13% *p* = 0.052	UA Non-OB: 2.2% OB: 2.9% CA Non-OB: 11.6% OB: 15.4% *p* = 0.191	UA Non-OB: 1.4% OB: 1.8% *p*: 0.455 CA Non-OB: 4.3% OB: 3% *p*: 0.437	NI	OT: Longer in OB patients. No differences regarding overall complications, wound complications, and readmissions
Timmerman et al. [28]	Open: NI CL: NI	NI	NI	UW: 4.5 (d) NW: 3.0 (d) OW: 2.0 (d) OB: 3.5 (d) UW vs. NW, *p*: 0.001 OB vs. NW: *p* < 0.001	NI	UW: 25% NW: 15% OW: 9% OB: 25% UW vs. NW *p* = 0.041	NI	NI	UW children are at greater risk of misdiagnosis of AA, longer LOS, and increased postoperative complications than NW. OB children have longer LOS than NW
Litz et al. [29]	SP	No conversion	NW/OW/OB *p* = 0.514	NW/OW/OB *p* = 0.214	SSI NW/OW/OB *p*: 0.130 Organ space NW/OW/OB *p*: 0.725	NI	NW/OW/OB *p*: 0.967 ED visits NW/OW/OB *p* = 0.726	NI	Obesity does not impact on outcomes after SP
Witt et al. [30]	CL: 90.12% Open: 9.8%	NI	NW/OW/OB/ Morbidly obese *p* < 0.001	NW/OW/OB/ Morbidly obese *p* = 0.002	NW/OW/OB/ Morbidly obese Superficial wound infection *p* = 0.03	Overall: 4.96% NW: 4.7% OW: 5.27% Obese: 5.73% Morbidly obese: 7.26% *p* = 0.01	NW/OW/OB/ Morbidly obese *p* = 0.16	Death within 30 days: NW: 0.02% OW/OB/ Morbidly obese: 0.00% *p* =1.00	Increased OT, LOS, SSI, and overall complications with increased BMI category
Mohan et al. [31]	SP	Conversion from SP to CL: 6	(median) UW/NW: 39 OW: 41 OB: 39 *p* = 0.43	UW/NW: 18 OW: 16 OB: 20 *p* = 0.13	UW/NW: 4% OW : 7% OB: 11% *p* = 0.33	NI	NI	NI	SP appendectomy has a significantly lower OT in all groups
Delgado-Miguel et al. [32]	Open: NW/OW: 237/44 CL: NW/OW: 69/57	NI	NW: 44.6 ± 18.2 OW: 57.6 ± 22.5 *p* < 0.001	NW: 3.29 ± 2.87 days OW: 3.43 ± 2.75 days *p*: 0.344	Wound infection NW/OW *p* < 0.001 Wound dehiscence NW/OW *p* < 0.001	NI	NI	NI	OW had longer OT and higher risk of wound infection and wound dehiscence
Lorio et al. [33]	NI	NI	NI	Non-OB/OB *p* = 0.54	NI	NI	NI	NI	No differences in LOS between non-OB and OB patients
Papillon et al. [34]	CL	No conversion	NW/OW/OB: *p*: 0.463	Median: 2 days NW/OW/OB: *p*: 0.174	NI	NI	NW/OW/OB: *p*: 0.352	NI	BMI does not influence (a) OT; (b) LOS; (c) Adverse events.

OT, Operative Time; LOS, Length of Stay; SSI, Surgical Site Infection; CL, conventional laparoscopy; OB, Obese; MOB, moderately obese; VOB: very obese; NI, No Information; NW, Normal Weight; CL, Conventional Laparoscopy; OW, Overweight; SP, Single port; CA, Complicated Appendicitis; ED, Emergency Department; AA, Acute Appendicitis; SIPES, Single-Incision Pediatric Endo-surgery; UW, Underweight; UA, Uncomplicated Appendicitis.

## Data Availability

Data sharing is not applicable.
